# Ferroptosis and Acute Kidney Injury (AKI): Molecular Mechanisms and Therapeutic Potentials

**DOI:** 10.3389/fphar.2022.858676

**Published:** 2022-04-19

**Authors:** Qi Feng, Xiaoyue Yu, Yingjin Qiao, Shaokang Pan, Rui Wang, Bin Zheng, Hui Wang, Kai-Di Ren, Hui Liu, Yang Yang

**Affiliations:** ^1^ Research Institute of Nephrology, Zhengzhou University, The First Affiliated Hospital of Zhengzhou University, Zhengzhou, China; ^2^ Department of Integrated Traditional and Western Nephrology, The First Affiliated Hospital of Zhengzhou University, Zhengzhou, China; ^3^ Henan Province Research Center for Kidney Disease, The First Affiliated Hospital of Zhengzhou University, Zhengzhou, China; ^4^ Blood Purification Center, The First Affiliated Hospital of Zhengzhou University, Zhengzhou, China; ^5^ Department of Pharmacy, The First Affiliated Hospital of Zhengzhou University, Zhengzhou, China; ^6^ School of Laboratory Medicine, Xinxiang Medical University, Xinxiang, China; ^7^ Clinical Systems Biology Laboratories, The First Affiliated Hospital of Zhengzhou University, Zhengzhou, China

**Keywords:** ferroptosis, acute kidney injury (AKI), mechanisms, regulators, treatment progress

## Abstract

Acute kidney injury (AKI), a common and serious clinical kidney syndrome with high incidence and mortality, is caused by multiple pathogenic factors, such as ischemia, nephrotoxic drugs, oxidative stress, inflammation, and urinary tract obstruction. Cell death, which is divided into several types, is critical for normal growth and development and maintaining dynamic balance. Ferroptosis, an iron-dependent nonapoptotic type of cell death, is characterized by iron overload, reactive oxygen species accumulation, and lipid peroxidation. Recently, growing evidence demonstrated the important role of ferroptosis in the development of various kidney diseases, including renal clear cell carcinoma, diabetic nephropathy, and AKI. However, the exact mechanism of ferroptosis participating in the initiation and progression of AKI has not been fully revealed. Herein, we aim to systematically discuss the definition of ferroptosis, the associated mechanisms and key regulators, and pharmacological progress and summarize the most recent discoveries about the role and mechanism of ferroptosis in AKI development. We further conclude its potential therapeutic strategies in AKI.

## Introduction

**FIGURE 1:**
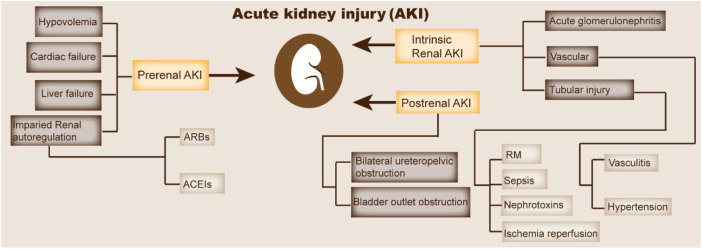
Major pathogenesis of AKI. This figure indicates the pathophysiology and etiology of AKI. CHF, congestive heart failure; ARBs, angiotensin receptor blockers; ACEIs, angiotensin-converting enzyme inhibitors; RM, rhabdomyolysis.

Acute kidney injury (AKI), which is characterized by a rapid decline in renal function, is caused by various physiological and pathological factors (Figure 1) (Kellum et al., 2021). In recent years, the incidence and mortality rates of AKI have remained high and exhibit an annual increasing trend (Liu et al., 2020). According to epidemiological investigation, the incidence rate of AKI is increasing with the increase of the aging population, the incidence rate of AKI in general inpatients is 5%, and the mortality rate of severe patients is over 50% (Ronco et al., 2019). The main clinical manifestation of AKI is the sudden or continuous decline of renal function in a short time (several hours to weeks), resulting in the accumulation of harmful and toxic metabolites in the body, and severe patients may also progress to renal failure or even death (Deep et al., 2021).

Given the pathogenesis of AKI, oxidative stress, inflammation, hypoxia, and apoptosis caused by surgery, rhabdomyolysis (RM), infection, ischemia–reperfusion injury (IRI), sepsis, and nephrotoxic drugs are the main causes (Ow et al., 2021). In clinical treatment, although blood purification can improve renal function after AKI, it cannot completely cure the disease. Relevant studies have also shown that the occurrence of AKI increases the potential risk of patients developing chronic kidney disease (CKD) and end-stage renal disease.

Cell death is critical for maintaining dynamic balance in the body. In recent years, other forms of cell death besides apoptosis, including ferroptosis, necroptosis, and pyroptosis, have been gradually recognized with the deepening of research (D'Arcy, 2019). Direct evidence shows that ferroptosis inhibitors possess renal protective effects in various animal models of AKI, suggesting that ferroptosis plays an important role in the initiation and progression of AKI (Hu et al., 2019). Therefore, it is of great significance to explore the mechanism of ferroptosis in AKI and clarify the effects of ferroptosis on the prognosis of AKI to CKD.

## Overview of Ferroptosis

Iron is an indispensable trace element in the human body, which plays multiple important biological functions, including the induction of ATP production, synthesis of DNA and heme, and many other physiological activities (Milto et al., 2016). Intracellular iron content, especially ferrous iron overload, could induce lipid peroxidation. In 2012, Dixon et al. officially named this iron-dependent cell death mediated by excessive lipid peroxidation as ferroptosis for the first time. Intracellular iron retention, reduced glutathione (GSH) content, and lipid reactive oxygen species (ROS) accumulation are the main characteristics of ferroptosis (Dixon et al., 2012). The excessive accumulation of ROS can activate intracellular oxidative stress response and damage proteins, nucleic acids, and lipids, finally resulting in the occurrence of ferroptosis (Tang et al., 2021). Ferroptosis is different from the previously found regulatory cell death patterns such as apoptosis, necrosis, pyroptosis, and autophagy in morphology, genetics, and mechanisms (Hirschhorn and Stockwell, 2019) (Table 1).

**TABLE 1:** Comparison between ferroptosis and other types of cell death.

Type of Cell Death	Definition	Morphological Features	Biochemical Features	Immune Features	Regulatory pathways	Key Genes
Ferroptosis	Non-apoptotic cell death characterized by iron-dependent lipid peroxidation	Small mitochondria with increased mitochondrial membrane densities, reduction or vanishing of mitochondria crista, outer mitochondrial membrane rupture and normal nucleus	Iron accumulation and lipid peroxidation	Promote inflammation	Xc^−^-GPX4, MVA, HSPB1-TFR1, p62-Keap1-Nrf2, p53-SLC7A11, ATG5-ATG7-NCOA4, p53-SAT1-ALOX15, FSP1-COQ10-NAD(P)H, RPL8, HSPB1, CISD1, LSH, EGLN, FANCD2, CHAC1	GPX4, TFR1, SLC7A11, Nrf2, NCOA4, p53, HSPB1, ACSL4, FSP1
Apoptosis	Autonomous and orderly death of genetically controlled cells to maintain internal stability	Cellular and nuclear volume reduction, chromatin agglutination, nuclear fragmentation, formation of apoptotic bodies and cytoskeletal disintegration, no significant changes in mitochondrial structure	DNA fragmentation decreases the mitochondrial membrane potential	Inhibit inflammation	Death receptor, mitochondrion and endoplasmic reticulum pathways, caspase, p53, Bcl-2	Caspase, Bcl-2, Bax, p53, Fas
Necroptosis	A mode of cell death that begins with a necrotic phenotype in the form of apoptosis	Plasma membrane break down, generalized swelling of the cytoplasm and organelles, moderate chromatin condensation, spillage of cellular constituents into microenvironment	Enrichment of kinase and drop in ATP level	Promote inflammation	TNF, RIP1/RIP3-MLKL; SIRT5, Toll-like receptors, PKC-MAPK-AP-1, ROS-related metabolic pathway	ATG5, ATG7, LC3, Beclin-1, DRAM3, TFEB
Pyroptosis	Pyrooptin-mediated programmed cell necrosis dependent on inflammatory caspase activation	Loss of membrane integrity, loss of organelles DNA condensation and fragmentation	Formation of inflammasomes, activation of caspase-1, release of pro-inflammatory factors	Promote inflammation	Caspase-1, NLRP3-mediated pathway	Caspase-1, IL-1β, IL-18
Autophagy	Under the regulation of related genes, the process by which lysosomes degrade cell’s own damaged organelles and macromolecular substances	Formation of double-membraned autolysosomes, including macro autophagy, micro autophagy, and chaperone-mediated autophagy	Increased lysosomal activity	Promote inflammation	mTOR, Beclin-1, ATG, ULK1, PI3K, p53	RIP1, RIP3

ACSL4, acyl-CoA synthetase long-chain family member 4; ALOX-15, arachidonate lipoxygenase 15; AP-1, activator protein-1; ATG5, autophagy-related 5; ATG7, autophagy-related 7; COQ10, coenzyme Q10; DRAM3, damage regulated autophagy modulator 3; FSP1, ferroptosis suppressor protein 1; GPX4, glutathione peroxidase 4; HSP*β*1, heat shock protein beta-1; Keap1, Keleh-like ECH-associated protein 1, MAPK, mitogen-activated protein kinase; MLKL, mixed lineage kinase domain like protein; mTOR, mammalian target of rapamycin; MVA, mevalonate; LC3, microtubule-associated protein 1 light chain3; NCOA4, nuclear receptor coactivator 4; Nrf2, nuclear factor erythroid 2-related factor 2; PKC, protein kinase C; RIP, receptor-interacting serine/threonine kinase; ROS, reactive oxygen species; SAT1, spermidine/spermine N1-acetyltransferase 1; SLC7A11, solute carrier family 7 member 11; system Xc^−^, cysteine/glutamate transporter receptor; TFEB, transcription factor EB; TFR1, transferrin receptor 1; TNF-α, tumor necrosis factor *α*.

A number of studies have investigated ferroptosis, however, the specific mechanisms of ferroptosis are still unknown. With the deepening of research on ferroptosis, various regulators and mechanismas have been developed successively, which provides new insights into the treatment of ferroptosis-associated diseases.

## Mechanisms and Key Regulators of Ferroptosis

**FIGURE 2:**
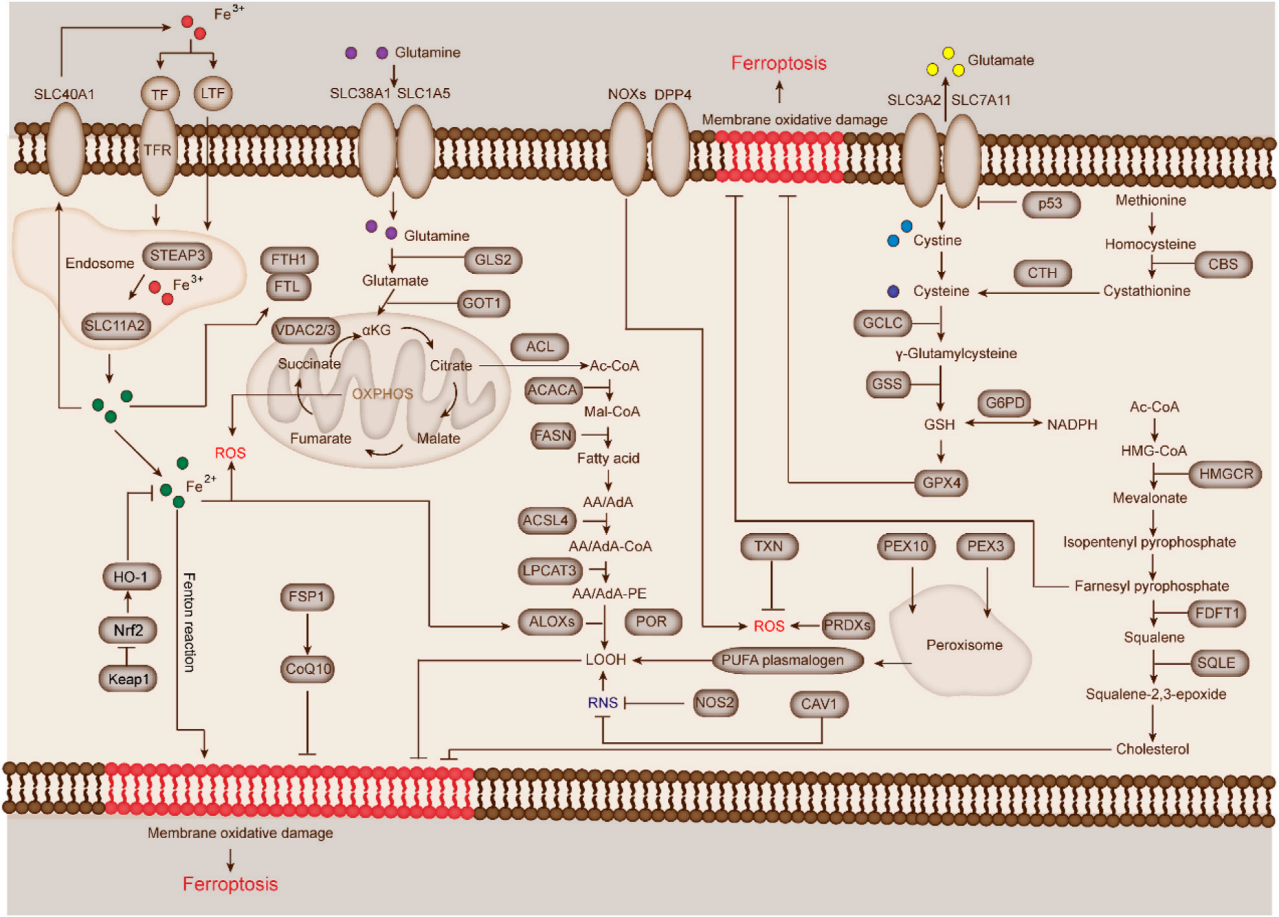
Mechanisms and key regulators of ferroptosis. The major mechanisms and key regulators of ferroptosis can be roughly divided into two important mechanisms (iron metabolism and lipid peroxidation) and seven key regulators (system Xc^−^, GPX4, p53, acyl-CoA synthetase long-chain family member 4 [ACSL4], FSP1, and Nrf2). In addition, other mechanisms and regulators, such as p53/SLC7A11 and VDAC2/3 are also involved in lipid regulation and ferroptosis.

During the past few years, the process and functions of ferroptosis has been well studied. In addition, several regulators of ferroptosis have been extensively investigated, including system Xc^−^, GPX4, p53, FSP1, and nuclear factor erythroid 2-related factor (Nrf2). In this part, we will briefly describe the major regulators and related molecular mechanisms of ferroptosis (Figure 2).

### Iron Metabolism

As an important inducer of ROS production through enzymatic or non-enzymatic reactions, iron plays an important role in ferroptosis. In general, under normal conditions, extracellular Fe^3+^ ions first bind to transferrin (TFR), and then are transported into cells via membrane transferrin receptor 1 (TFR1) and stored in the form of iron protein complex (mainly ferritin). Fe^3+^ ions are reduced to Fe^2+^ and subsequently transported and stored in the cell iron pool, while excess Fe^2+^ ions are stored in ferritin (van Swelm et al., 2020). Ferritin is an iron storage protein complex, which is composed of ferritin light chain and ferritin heavy chain 1 (FTH1). In the case of iron metabolic disorder, the low expression of FTH1 and the overexpression of TFR1 often lead to the excessive accumulation of Fe^2+^ ions, which induce the production and accumulation of a large amount of ROS through Fenton reaction and eventually promote cell ferroptosis (Daher and Karim, 2017). TFR1 has been considered as the only known protein that controls iron input in mammalian cells and indispensable in iron metabolism (Kawabata, 2019).

### Lipid Peroxidation

Lipid peroxidation plays a driving role in the occurrence of ferroptosis, which can be completed by non-enzymatic or enzymatic reaction. Compared with unsaturated fatty acids and monounsaturated fatty acids, polyunsaturated fatty acids (PUFAs) are more prone to lipid peroxidation and ferroptosis (Latunde-Dada, 2017). The abundance and location of PUFAs determine the degree of lipid peroxidation and ferroptosis. Free PUFAs are substrates for the synthesis of lipid signal transduction mediators, but they must be esterified into membrane phospholipids and oxidized to initiate ferroptosis. The formation of PUFA coenzyme A derivatives is a necessary condition for the generation of ferroptosis, and the involvement of regulatory enzymes in the biosynthesis of PUFAs in membrane phospholipids can trigger or prevent ferroptosis (Yang et al., 2016).

ACSL4 and lysophosphatidylcholine acyltransferase 3 are involved in the biosynthesis of phosphatidylethanolamine in the cell membrane. The deletion of these genes will increase the cell resistance to ferroptosis. By contrast, cells supplemented with arachidonic acid or other PUFAs increases their sensitivity to ferroptosis (Doll et al., 2017; Shimbara-Matsubayashi et al., 2019). Lipoxygenases (LOXs) are important enzyme systems that mediate the formation of ferroptosis peroxides. Free PUFAs are the preferred substrate of LOX; knocking out LOXs can alleviate the injury caused by ferroptosis. In addition, phosphatidylethanolamine can be further oxidized under the catalysis of LOXs, thereby inducing cell ferroptosis (Stockwell et al., 2017).

### System Xc^−^

System Xc^−^, a cystine glutamate transporter that widely expressed on the cell membrance, is composed of light chain subunit (SLC7A11) and heavy chain subunit (SLC3A2) (Dixon et al., 2012). Cystine and glutamate are exchanged at a ratio of 1:1 inside and outside the cell via system Xc^−^. System Xc^−^ transfers cystine or cystine sulfide into cells and reduces it to cysteine (Chen et al., 2021a). Under extracellular oxidation, the exchange of cystine/cystine sulfide and glutamate is the upstream of ferroptosis (Hirschhorn and Stockwell, 2019). The exchange of extracellular cystine and intracellular glutamate affects the synthesis of GSH and maintains the stability of intracellular GSH, thereby protecting cells from oxidative stress (Bersuker et al., 2019).

GSH is mainly composed of glutamate, cysteine, and glycine, and the sulfhydryl structure of GSH can be oxidized and dehydrogenated, making GSH an important antioxidant and free radical scavenger in the body (Bannai and Tateishi, 1986). GSH is an essential cofactor for GPXs to exert antioxidant function (Lash, 2005). Reduced GSH is an important intracellular antioxidant in mammals. Glutamic acid, cysteine, and glycine generate GSH in two steps under the catalysis of glutamyl cysteine ligase and glutathione synthase (Bjørklund et al., 2021). Therefore, the availability of glutamate and cysteine affects GSH biosynthesis. Recently, studies have found that ferroptosis induced by erastin and sulfasalazine can inhibit system Xc^−^, reduce intracellular cystine uptake, decrease GSH synthesis, induce lipid peroxide accumulation, and finally lead to cell ferroptosis (Floros et al., 2021). Jiang et al. reported that pachymic acid could alleviate IRI-induced AKI (IRI-AKI) by upregulating Nrf2, SLC7A11, and HO-1 expression, which indicated the importance of system Xc^−^ in kidney injury (Jiang et al., 2021). Tumor suppressor protein p53 plays a critical role in cellular response to various life activities, such as DNA damage, hypoxia, oncogene activation, and ferroptosis (Kang et al., 2019). p53 downregulates SLC7A11 and inhibits the uptake of cysteine via system Xc^−^, thus affecting GPX4 activity, resulting in renal cell ferroptosis (Jiang et al., 2015a). Li et al. showed that *α*-lipoic acid ameliorates folic acid (FA)-induced AKI by inhibiting p53 and up-regulating SLC7A11 expression (Li et al., 2021a). Additionally, Beclin1, a key regulator of autophagy, has been proved to be a new driver of ferroptosis and can promote ferroptosis by regulating the action of system Xc^−^ in tumor cells (Kang et al., 2018).

In summary, system Xc^−^ can maintain GSH levels by maintaining the balance between intracellular and extracellular cystine and glutamate. Once the equilibrium state is broken, the depletion of intracellular GSH level will decrease the synthesis and activity of GPX4, finally resulting in the occurrence of cell ferroptosis.

### GPX4

GPXs, one family of antioxidant enzymes that are widely expressed in different tissues and can reduce peroxides (Jiao et al., 2017). The GPXs contains eight members, however, onlyGPX1, GPX3, and GPX4 are found to be expressed in the kidney; notably, GPX4 plays a more important role in ferroptosis (Brigelius-Flohé and Maiorino, 2013). Ursini et al. firstly isolated GPX4 from pig liver in 1982 and found that it had the ability to inhibit iron-triggered lipid peroxidation in microsomes (Ursini et al., 1982). At present, GPX4 is the only known enzyme that can directly remove lipid peroxidation, which can reduce harmful lipid peroxidation into harmless substances, so as to interrupt the occurrence of lipid peroxidation and finally inhibit cell ferroptosis (Forcina and Dixon, 2019). The inhibition of GPX4 activity can lead to lipid peroxide accumulation, which is a marker of ferroptosis (Seibt et al., 2019).

Chemical compounds such as RSL3, DPI7, and DPI10 can directly act on GPX4 and block its activity, thus leading to the inhibition of cellular antioxidant capacity and ROS accumulation and finally resulting in the development of ferroptosis (Liang et al., 2019). Yang et al. found that GPX4 was a key regulator of ferroptosis and inhibiting GPX4 could induce tumor cell ferroptosis (Yang et al., 2014). Gaschler et al. found that five-membered cyclic peroxides 1 and 2 could increase the sensitivity of cells to ferroptosis by indirectly inactivating GPX4 (Gaschler et al., 2018). Kinowaki et al. found that GPX4 was significantly correlated with the prognosis of diffuse B-cell lymphoma and that GPX4-depleted cells were sensitive to ROS-induced cell ferroptosis (Kinowaki et al., 2018). The mechanism of ferroptosis induced by erastin and RSL3 is not exactly the same. Erastin mainly inhibits system Xc^−^, thus suppressing GSH synthesis, but indirectly inhibiting GPX4, which results in cell ferroptosis. By contrast, RSL3 can directly bind to GPX4, resulting in cell lipid peroxidation and ROS accumulation and finally leading to cell ferroptosis (Friedmann Angeli et al., 2014).

### p53

p53 is an important tumor suppressor. Aside from its effects on cell death, autophagy, apoptosis, and pyroptosis, p53 is also involved in regulating cell ferroptosis. p53 can promote ferroptosis by decreasing the expression of SLC7A11. It also suppresses ferroptosis via the direct downregulation of dipeptidyl peptidase 4 (DPP4) or upregulation of cyclin dependent kinase inhibitor 1A/p21 expression. For example, Jiang et al. firstly found that p53 inhibited the uptake of cystine by blocking SLC7A11, which caused a significant reduction of GSH, and induction of cell ferroptosis (Jiang et al., 2015a). Chu et al. revealed that knockout of arachidonate 12-lipoxygenase (ALOX12) specifically blocked the occurrence of ferroptosis, which proved that p53 indirectly activated the function of ALOX12 by inhibiting SLC7A11, resulting in ALOX12-dependent ferroptosis after ROS stress (Chu et al., 2019). Xie et al. demonstrated that p53 could inhibit the ferroptosis sensitivity of tumor cells induced by erastin by blocking DPP4 activity (Xie et al., 2017). In conclusion, p53 can not only regulate apoptosis and cell cycle arrest, but also inhibit the occurrence and development of tumors by regulating ferroptosis.

### FSP1

FSP1, previously known as apoptosis-inducing factor mitochondrion-associated 2, has been recently proved as an endogenous ferroptosis inhibitor (Doll et al., 2019). Studies demonstrated that FSP1 was a key component of the non-mitochondrial coenzyme Q (CoQ) antioxidant system and could promote the generation of lipophilic free radicals and the recruitment of antioxidants (RTA) by decreasing the expression of coenzyme Q10. RTA can effectively relieve ferroptosis by neutralizing the accumulation of lipid peroxide (Santoro, 2020). Therefore, FSP1 is considered as a ferroptosis suppressor in various cancer cells. Bersuker et al. recently found that when GPX4 was inactivated, FSP1 could maintain the growth of lung cancer cells, which implied that FSP1 contributed to the resistance against cell ferroptosis and might be one important drug target for cancer treatment (Bersuker et al., 2019). Additionally, through high-throughput screening among 10,000 small molecule drugs, researchers found that the inhibitor iFSP1 could significantly upregulate the sensitivity of tumor cells to ferroptosis by inhibiting FSP1. Meanwhile, Dai et al. reported that FSP1 could block the occurrence of cell ferroptosis by affecting ubiquinol metabolism (Dai et al., 2020).

### Nrf2

Nrf2 is an important regulator of cellular antioxidant responses (Baird and Yamamoto, 2020).

Studies have reported that Nrf2 can transcriptionally regulate the expression of a series of downstream genes related to ferroptosis, including NAD(P)H:quinone oxidoreductase 1, HO-1, SLC7A11, and GPX4 (Abdalkader et al., 2018). Thus, Nrf2 is considered as an important negative regulator of ferroptosis. Fan et al. reported that overexpression of Nrf2 could up-regulate SLC7A11, thereby inhibiting the cell ferroptosis (Fan et al., 2017). Tian et al. found Nrf2 attenuated myocardial ischemia-reperfusion injury in diabetic rats via blocking ferroptosis (Tian et al., 2022). In addition, Sun et al. reported that the p62/Keap1/Nrf2 axis was activated under the stimulation of ferroptosis inducers, causing the activation and transcription of HO-1 and FTH-1 and finally decreasing the sensitivity to ferroptosis in cells (Sun et al., 2016). Additionally, Nrf2 directly or indirectly regulates the expression and function of GPX4 (Hayes and Dinkova-Kostova, 2014). Liao et al. showed that DJ-1 could inhibit trophoblast ferroptosis of preeclampsia by upregulating the expression of Nrf2 and its downstream gene *GPX4* (Liao et al., 2022). Ge et al. demonstrated that Zinc attenuated contusion spinal cord injury by inhibiting ferroptosis via activating Nrf2/GPX4 pathway (Ge et al., 2021). On the contrary, during the past few years, it was found that Nrf2 activation could induce ferroptosis. For example, Wei et al. revealed that Tagitinin C promoted cell ferroptosis in colorectal cancer by activating PERK/Nrf2/HO-1 pathway (Wei et al., 2021). Chen et al. found *Nrf2* was the downstream gene of tumor suppressor ARF, and ARF could induce ferroptosis of tumor cells by inhibiting Nrf2 activation, which uncovered that Nrf2 activation might be a positive regulator in ferroptosis (Chen et al., 2017). Nrf2 activation could upregulate the expression of HO-1, however, several studied supported that HO-1 overexpression would trigger ferroptosis by promoting iron overloading and lipid peroxidation (Xu et al., 2019). Therefore, these studies suggested a dual role of Nrf2 in ferroptosis.

Recently, Nrf2 was also proved to participate in regulating ferroptosis in kidney injury. Li et al. reported that upregulating Nrf2 could rescue kidney injury and renal failure by inhibiting ferroptosis in diabetic mice (Li et al., 2021b). Meanwhile, it found that roxadustat treatment could attenuate FA-induced AKI by blocking ferroptosis through regulating Nrf2 (Li et al., 2020a). Meng et al. discovered that the activation of Nrf2 by ADAMTS-13 could inhibit ferroptosis to ameliorate cisplatin-induced AKI (Meng et al., 2021). Jiang et al. found that pachymic acid exerted a protective effect on IRI-induced AKI mice through the inhibition of ferroptosis by activating Nrf2 (Jiang et al., 2021). Yang et al. demonstrated that dimethyl fumarate could prevent ferroptosis to attenuate AKI by upregulating Nrf2 (Yang et al., 2021). Deng et al. revealed that the inhibition of mitochondrial iron overload could alleviate aristolactam I-induced AKI by regulating the Nrf2/HO-1/GPX4 axis (Deng et al., 2020). The above studies showed that Nrf2 played a key role in renal injury caused by ferroptosis. Since the specific mechanism of Nrf2-mediated ferroptosis in AKI renal injury has not been fully clarified, future work should focus on identifying the target gene of Nrf2 and further clarify the mechanism of this gene in specifically regulating ferroptosis in AKI, which is of great significance for the development of therapeutic drugs for AKI in the future.

### ACSL4

ACSL4 is a member of the long-chain acyl coenzyme A synthetase family, which can catalyze the activation of fatty acids to synthesize acyl coenzyme A. Therefore, it is considered as a key enzyme in fatty acid catabolism (Qu et al., 2021). Different from other ACSL family members, ACSL4 can activate long-chain PUFAs and participate in the synthesis of membrane phospholipids. Meanwhile, knockout of ACSL4 will cause ferroptosis (Doll et al., 2017).

The long-chain PUFAs on these membranes are easily oxidized, and ferroptosis inducers such as RSL3 can induce cell ferroptosis. ] ACSl4^−/−^ knockout cells displayed resistance against ferroptosis, and the inhibitory effect of lipid peroxide on GPX4 required the participation of ACSL4 (Shimbara-Matsubayashi et al., 2019). Doll et al. found that thiazolidinediones could protect ACSL4 knockout embryonic fibroblasts, reduce the degree of membrane lipid oxidation and cell death, and significantly prolong the survival time of ACSL4 knockout mice (Doll et al., 2017). Wang et al. demonstrated thaterastin and RSL3 induced renal tubular cell death accompanied by high ACSL4 levels *in vitro*, while the ACSL4 inhibitor rosiglitazone improved the survival rate and alleviated kidney injury in diabetic nephropathy mice, and decreased lipid peroxidation products and iron content (Wang et al., 2020). Li et al. reported that ACSL4 played a critical role in ferroptosis-mediated tissue injury in IR mouse model, and special protein 1 could increase ACSL4 transcription and further promote the progression of ferroptosis (Li et al., 2019a). Feng et al. revealed that miRNA-17-92 exerted protective effects on endothelial cells by inhibiting erastin-induced ferroptosis via targeting the A20/ACSL4 pathway (Xiao et al., 2019a). Cui et al. showed that ACSL4 induced neuronal death by increasing lipid peroxidation and promoted ischemic stroke by enhancing ferroptosis-induced brain injury and neuroinflammation (Cui et al., 2021). These studies suggested that ACSL4 might be a crucial determinant of ferroptosis (Yuan et al., 2016).

### Other Mechanisms

Several other pathways might also be closely related to the occurrence of ferroptosis. Voltage-dependent anion channels (VDACs), a type of channel proteins located in the mitochondrial outer membrane, play an essential role in the communication between mitochondria and other organelles (Skonieczna et al., 2017). Yagoda et al. revealed that erastin could directly combine with VDAC2/3 to reduce the oxidation rate of NADH, and induce cell ferroptosis (Yagoda et al., 2007). In addition, the PI3K/Akt/mTOR (Yi et al., 2020), p53/SLC7A11 (Jiang et al., 2015b), and glutamine metabolic pathways (Gao et al., 2015) play negative regulatory roles in the occurrence of ferroptosis. To sum up, the research on ferroptosis is still in the preliminary stage, and the specific mechanisms have not been completely identified.

## Pharmacological Progress of Ferroptosis

In recent years, numerous natural and synthetic drugs related to ferroptosis, including inducers and inhibitors, have been identified. Generally, for ferroptosis in non-neoplastic diseases (e.g., AKI, CKD), ferroptosis inhibitors can be used to reduce iron levels, restrain lipid peroxidation, and relieve renal function insufficiency. For carcinoma, cell death can be promoted by ferroptosis inducers to block tumor cell proliferation by increasing ROS levels and inducing the progression of iron-dependent lipid peroxidation (Xie et al., 2016) (Table 2).

**TABLE 2:** Inducers and inhibitors of ferroptosis.

Classification	Reagents	Targets	Mechanisms	References
Ferroptosis inducers	Sulphasalazine	System Xc^−^	Cysteine deprivation	Gout et al. (2001)
	Sorafenib	System Xc^−^	Cysteine deprivation	Louandre et al. (2013); Louandre et al. (2015)
	Erastin	System Xc^−^, VDAC2/3	Cysteine deprivation	Dolma et al. (2003); Yagoda et al. (2007)
	RSL3	GPX4	GPX4 inactivation and GSH deletion	Yang et al. (2014)
	ML162	GPX4	GPX4 inactivation and GSH deletion	Moosmayer et al. (2021)
	FINO_2_	GPX4	GPX4 inactivation and lipid peroxides accumulation	Gaschler et al. (2018)
	FIN56	CoQ10 and GPX4	CoQ10 deletion and GPX4 inactivation	Shimada et al. (2016)
	BSO	GHS	GHS deletion	Yang et al. (2014)
	DPI2	GSH	GHS deletion	Yang et al. (2014)
	Cisplatin	GSH	Decreased GSH levels and GPX4 inactivation	Yamaguchi et al. (2013)
	Statins	HMG	CoQ10 deletion	Chen et al. (2021b)
	Siramesine	Ferroportin	Increased cellular iron	Ma et al. (2016)
	lapatinib	Transferrin	Increased cellular iron	Ma et al. (2016)
Ferroptosis inhibitors	Ferrostatin-1	ROS from lipid peroxidation	Inhibition of lipid peroxidation	Skouta et al. (2014)
	Liproxstatin-1	ROS from lipid peroxidation	Inhibition of lipid peroxidation	Friedmann Angeli et al. (2014)
	Vitamin E	ROS from lipid peroxidation	Inhibition of lipid peroxidation	Hinman et al. (2018)
	SRS 16–86	ROS from lipid peroxidation	Inhibition of lipid peroxidation	Linkermann et al. (2014a)
	SRS 11–92	ROS from lipid peroxidation	Inhibition of lipid peroxidation	Skouta et al. (2014)
	TEMPO	Radical-trapping antioxidant	Elimination of oxygen free radicals	Griesser et al. (2018)
	PMC	Radical-trapping antioxidant	Elimination of oxygen free radicals	Shah et al. (2017)
	Tetrahydronapthy-ridinols	Radical-trapping antioxidant	Elimination of oxygen free radicals	Angeli et al. (2017)
	Phenothiazine	Radical-trapping antioxidant	Elimination of oxygen free radicals	Shah et al. (2017)
	Phenoxazine	Radical-trapping antioxidant	Elimination of oxygen free radicals	Shah et al. (2017)
	Diarylamine	Radical-trapping antioxidant	Elimination of oxygen free radicals	Shah et al. (2017)
	XJB-5-131	Nitroxide antioxidant	Elimination of nitrogen oxides	Krainz et al. (2016)
	Baicalein	Lipoxygenases	Inactivation of lipoxygenase	Yang and Stockwell, (2016)
	BW A4C	Lipoxygenases	Inactivation of lipoxygenase	Angeli et al. (2017)
	AA-861	Lipoxygenases	Inactivation of lipoxygenase	Yang and Stockwell, (2016)
	CDC	Lipoxygenases	Inactivation of lipoxygenase	Yang and Stockwell, (2016)
	PD146176	Lipoxygenases	Inactivation of lipoxygenase	Yang and Stockwell, (2016)
	NDGA	Lipoxygenases	Inactivation of lipoxygenase	Probst et al. (2017)
	Zileuton	Lipoxygenases	Inactivation of lipoxygenase	Yang and Stockwell, (2016)
	Ciclopirox olamine	Intracellular iron	Decreased cellular iron	Dixon et al. (2012)
	2,2′-bipyridyl	Intracellular iron	Decreased cellular iron	Dixon et al. (2012)
	Deferoxamine mesylate	Intracellular iron	Decreased cellular iron	Yang and Stockwell, (2008)
	Deferoxamine	Intracellular iron	Decreased cellular iron	Dixon et al. (2012)

## Ferroptosis is Involved in the Development of AKI

AKI is a disease featured by acute renal insufficiency, which is caused by many factors. Although significant progress has been made, no specific drugs have been developed for the prevention and treatment of AKI (Kellum et al., 2021). Recent findings revealed that ferroptosis is a promising therapeutic target, especially in diseases dominated by kidney tubular injury (Carney, 2021). Therefore, studies on RM-, ischemia-reperfusion-, sepsis-, and nephrotoxic drug-induced AKI animal models have provided direct evidence to confirm the participation of ferroptosis in AKI.

### Ferroptosis and AKI Caused by RM

RM refers to a clinical syndrome caused by the entry of a large number of intracellular components such as myoglobin (Mb), phosphocreatine kinase, and lactate dehydrogenase into the peripheral blood, which accounts for 13%–50% of cases with AKI (Cabral et al., 2020). The accumulation of Mb in kidney is the core factor leading to kidney injury, and Mb-mediated lipid peroxidation in renal tubular epithelial cells is closely related to glutamate metabolism and can mediate proximal tubular ferroptosis (Vanholder et al., 2021). Guerrero-Hue et al. confirmed that ferroptosis was involved in the development of RM-associated AKI, and curcumin could alleviate RM-induced kidney injury via inhibiting ferroptosis of renal tubular epithelial cells (Guerrero-Hue et al., 2019). Zarjou et al. demonstrated that *FTH* ^−/-^ mice displayed a higher mortality and more serious kidney injury in RM-induced AKI model compared with wild-type mice, indicating that FTH exerted a protective effect on renal tubular injury in AKI (Zarjou et al., 2013).

### Ferroptosis and IRI-AKI

IRI refers to injury caused by the restoration of blood flow after the interruption or decline of blood flow in the body or organs for a period of time (Sharfuddin and Molitoris, 2011). Renal IRI is one of the main causes of AKI, and the pathophysiology of IRI-AKI includes mitochondrial dysfunction, inflammation, ROS, and lipid peroxidation (Malek and Nematbakhsh, 2015). Recent study demonstrated ferroptosis might be a new driver in initiating IRI-AKI (Yan et al., 2020). Huang et al. found that in an IRI-AKI mouse model, augmenter of liver regeneration (ALR) could regulate the development of ferroptosis through GSH/GPX4. Ding et al. reported that microRNAs also participated in the regulation of ferroptosis in an IRI-AKI mouse model (Ding et al., 2020). Additionally, Wang et al. showed ACSL4 knockout significanlty inhibited the ferroptosis of renal tubular epithelial cells in IRI-AKI mice, which indicated ACSL4 might be a key target for AKI treatment (Wang et al., 2022). XJB-5-131 is a new generation of antioxidant, which exerts dual effects of mitochondrial targeting and free radical scavenging. Zhao et al. confirmed that XJB-5-131 could specifically inhibit ferroptosis by inhibiting lipid peroxidation, so as to alleviate IRI-AKI (Zhao et al., 2020). Recently, the application of ferroptosis inhibitors, including Fer-1 and liproxstatin-1, had been found to exhibit protective effects against functional acute renal failure and structural organ damage in IRI-AKI mice (Yan et al., 2020). The latest study from Tonnus et al. showed that the deletion of FSP1 or GPX4 improved the sensitivity of tubular ferroptosis in IRI-AKI mice, which supported that the dysfunction of ferroptosis-surveilling systems enhanced the sensitivity of mice to tubular ferroptosis during AKI (Tonnus et al., 2021).

### Ferroptosis and Sepsis-Associated AKI

Sepsis refers to the systemic inflammatory responses caused by infection and can develop into severe sepsis, septic shock, and multiple organ dysfunction. Sepsis has been considered as one of the main causes of AKI, which is called SA-AKI (Bagshaw et al., 2009). The mechanism of SA-AKI is complicated, and the ideal therapeutic effects have not been achieved. A large number of studies revealed that renal tubular cell necrosis, apoptosis, and autophagy occur in SA-AKI, but these typical modes of cell death cannot fully explain the renal pathological changes in SA-AKI. Recently, Xiao et al. reported that ferroptosis of renal tubular epithelial cells was occurred in SA-AKI mice, and the results demonstrated that Maresin conjugates in tissue regeneration 1 (MCTR1) could significantly suppress cell ferroptosis in SA-AKI by activating Nrf2 pathway (Xiao et al., 2021). Zhang et al. demonstrated miR-124-3p.1 displayed inhibitory effect on the ferroptosis in SA-AKI by inhibiting the up-regulation of lysophosphatidylcholine acyltransferase 3, which indicated miR-124-3p.1 might be a biomarker and potential therapeutical target in AKI (Wu et al., 2022).

### Ferroptosis in Nephrotoxic Drug-Induced AKI

Nephrotoxic drugs (e.g., FA, cisplatin) is another key factor triggering AKI. FA at a high dosage might rapidly form crystals in renal tubules, resulting in AKI. Guo et al. found that lipid peroxidation occured in the kidney tissue of FA-induced AKI mouse model, and blocking Rev-erb-*α*/*β* could ameliorate FA-induced AKI by restraining ferroptosis (Guo et al., 2021). Martin-Sanchez et al. confirmed that treatment with the ferroptosis inhibitor Fer-1 could significantly alleviate renal function and reduce kidney damage in FA-induced AKI mice (Martin-Sanchez et al., 2017).

Cisplatin is a commonly used anticancer drug; however, it often causes nephrotoxicity, limiting its use. Previous studies had shown that the incidence of cisplatin-induced AKI was 20%–30%. Recently, studies showed that cisplatin administration in *FTH* knockout mice significantly induced proximal tubule injury compared with that in wild-type mice (Zarjou et al., 2013). Lu et al. reported that *Ras* homolog enriched in brain could relieve cisplatin-induced AKI by maintaining mitochondrial homeostasis (Lu et al., 2020). Zhou et al. recently reported that polydatin enabled to attenuate the cell ferroptosis in cisplatin-induced AKI via regulating system Xc−/GSH/GPX4 pathway and inhibiting iron metabolism disorders (Zhou et al., 2022). Deng et al. found Se/Albumin nanoparticles could alleviate cisplatin-induced AKI by inhibiting ferroptosis with a decrease of superoxide dismutase, and up-regulation of GSH and GPX4 (Deng et al., 2022).

## Biological Reactions Associated With Ferroptosis During AKI

**FIGURE 3:**
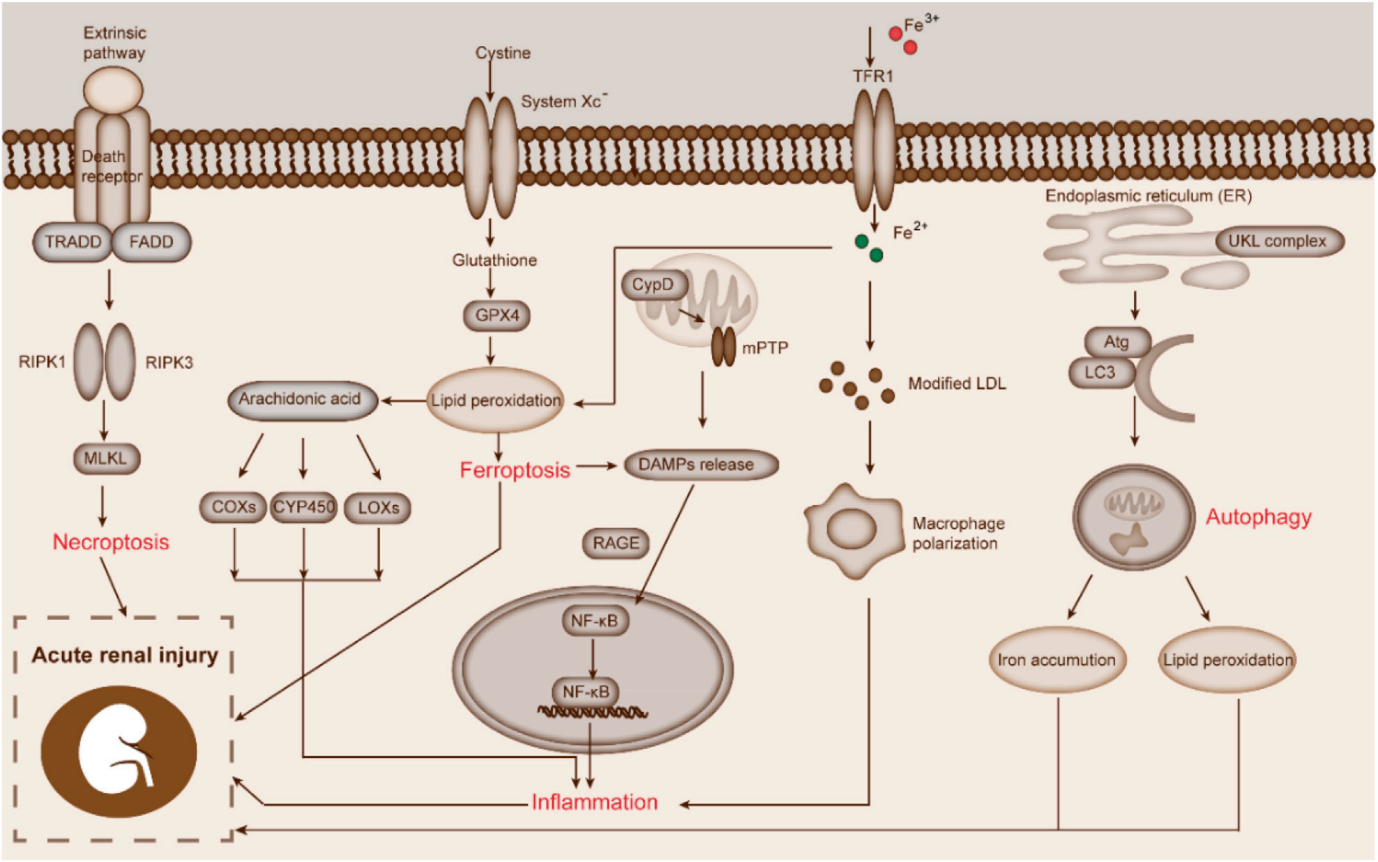
Biological responses correlated to ferroptosis during AKI. This figure displays the crosstalk between necroptosis, inflammation, autophagy, and ferroptosis in AKI.

Previous studies had shown that biological reactions such as necroptosis, inflammation, autophagy, and ferroptosis displayed a close relationship with the occurrence and development of various diseases in the human body. Therefore, in this part, we aim to summarize the recent studies about the crosstalk between various biological reactions and ferroptosis during AKI (Figure 3).

### Necroptosis

Among various types of cell death, necroptosis is a recently discovered and controllable programmed cell death mode. Generally, necroptosis is mediated by receptor-interacting serine/threonine protein kinase 3 and its substrate mixed lineage kinase domain-like protein (MLKL) (Chen et al., 2020). Several studies had shown that the imbalance of necroptosis was closely related to the development of various human diseases, such as inflammatory diseases, autoimmune diseases, tumors, kidney diseases, and degenerative diseases (Westman et al., 2019).

Since the discovery of ferroptosis and necroptosis, the role of necroptosis in the development of AKI and AKI to CKD progression had been deeply investigated. A growing body of evidence had described the essential roles of ferroptosis and necroptosis in AKI. Angeli et al. reported that necroptosis inhibitor protected the kidney injury from inhibiting necroptosis and ferroptosis in IRI- and cisplatin-induced AKI mice (Friedmann Angeli et al., 2014). Using *MLKI* ^−/−^ mice to establish IRI model, Müller et al. found that inhibiting necroptosis only exerted a slight protective effect on renal tubule injury and ferroptosis development. However, when ferroptosis inhibitors were used, they detected the high sensitivity of renal tubular epithelial cells to necroptosis, suggesting a “selective” interaction between necroptosis and ferroptosis (Müller et al., 2017). Martin-Sanchez et al. revealed that necroptosis-related proteins contributed to FA-induced AKI, but ferroptosis was still the main cause of FA-induced AKI. In addition, the secondary inflammatory responses triggered by ferroptosis further promoted renal injury in AKI, which suggested the association of ferroptosis and necroptosis to FA-induced AKI in mice (Martin-Sanchez et al., 2017).

### Autophagy

Autophagy is a biological process, in which cells wrap their cytoplasmic proteins or organelles to form autophagic vesicles, fuse with lysosomes, and degrade their inclusions, so as to realize metabolic requirements and organelle renewal in cells (Mizushima and Komatsu, 2011). Studies showed that autophagy was closely related to the initiation of cancers, neurodegenerative diseases, and kidney diseases (Luan et al., 2021). IRI, ROS, and inflammatory responses will affect autophagy, and the impairment of autophagy function will accelerate cell death (Mizushima and Levine, 2020). In mice models intervened by ferroptosis inducers, researchers observed the accumulation of autophagy lysosomes and the abnormal expression of autophagy-related genes such as *ATG3*, *ATG5*, and *Beclin1*. Interestingly, autophagy can trigger the occurrence of ferroptosis. Hou et al. found that knockout of autophagy-related genes limited the development of erastin-induced ferroptosis by down-regulating intracellular iron concentrations and lipid peroxidation (Hou et al., 2016). Masaldan et al. demonstrated that senescent cells were significantly resistant against ferroptosis, and autophagy activators could induce ferritin degradation, resulting in the down-regulation of TFR1 and ferritin (Masaldan et al., 2018). Additionally, Chen et al. revealed that legumain deficiency alleviated the development of renal tubular injury and ferroptosis in AKI mice, which proved legumain enhanced renal tubular epithelial cell ferroptosis by facilitating autophagy in AKI(Chen et al., 2021c).

### Inflammation

During ferroptosis, the shrinkage of cell and organelle membranes and the increase of permeability cause cell content release, including damage-associated molecular pattern (DAMPs) (Feng et al., 2020), which leads to local inflammatory responses (Mu et al., 2021). Li et al. reported that ferroptosis inhibition alleviated angiotensin II-induced inflammation and protected the brain from external injury by activating Nrf2/HO-1 pathway (Li et al., 2021c). In oxalate crystallization-induced AKI mouse mode, Proneth et al. observed necrotizing inflammatory response in tissues with ferroptosis, such as DAMPs release and production of proinflammatory factors. They also found that the inflammatory response could be effectively blocked by ferroptosis inhibitor (Proneth and Conrad, 2019). Linkermann et al. found the proinflammatory factor TNF-α released by macrophages exhibited side effects on the stability of ferroptosis-related protein GPX4 (Linkermann et al., 2014b). In addition, Li et al. observed that Fer-1 inhibited TLR4/Trif/type I IFN pathway and alleviated the inflammatory response in cardiac IRI mouse model (Li et al., 2019b). Guerrero-Hue et al. described that curcumin ameliorated kidney injury by decreasing inflammation-mediated cell ferroptosis (Guerrero-Hue et al., 2019).

### Other Metabolic Pathways

Recently, several other metabolic pathways closely related to ferroptosis were explored. Pannexin 1 is a channel protein that can release ATP and Su et al. found Pannexin 1 deficiency could reduce the ferroptosis of renal tubular cells in IRI-AKI mice by regulating the MAPK/ERK pathway and NOCA4-mediated ferritin autophagy (Su et al., 2019). CoQ, also known as ubiquinone, is a lipid-soluble quinone compound. CoQ produced by the mevalonate pathway is not only an antioxidant in cells, but also an effective inhibitor of ferroptosis. FIN56 could exhaust CoQ by regulating the activity of squalene synthase, leading to the accumulation of lethal lipid peroxide and occurrence of ferroptosis (Baschiera et al., 2021). Dixon et al. found that statins could deplete CoQ and enhance the cell sensitivity to ferroptosis by blocking 3-hydroxy-3-methylglutaryl coenzyme A reductase of the mevalonate pathway (Dixon et al., 2015). Besides, tremendous studies revealed that the p62/Keap1/Nrf2, Atg5/NCOA4, as well as glutamine metabolic pathway were also played key roles in ferroptosis by effectively regulating the intracellular iron content and ROS level (Li et al., 2020b).

## Treatment of AKI by Targeting Ferroptosis

**FIGURE 4:**
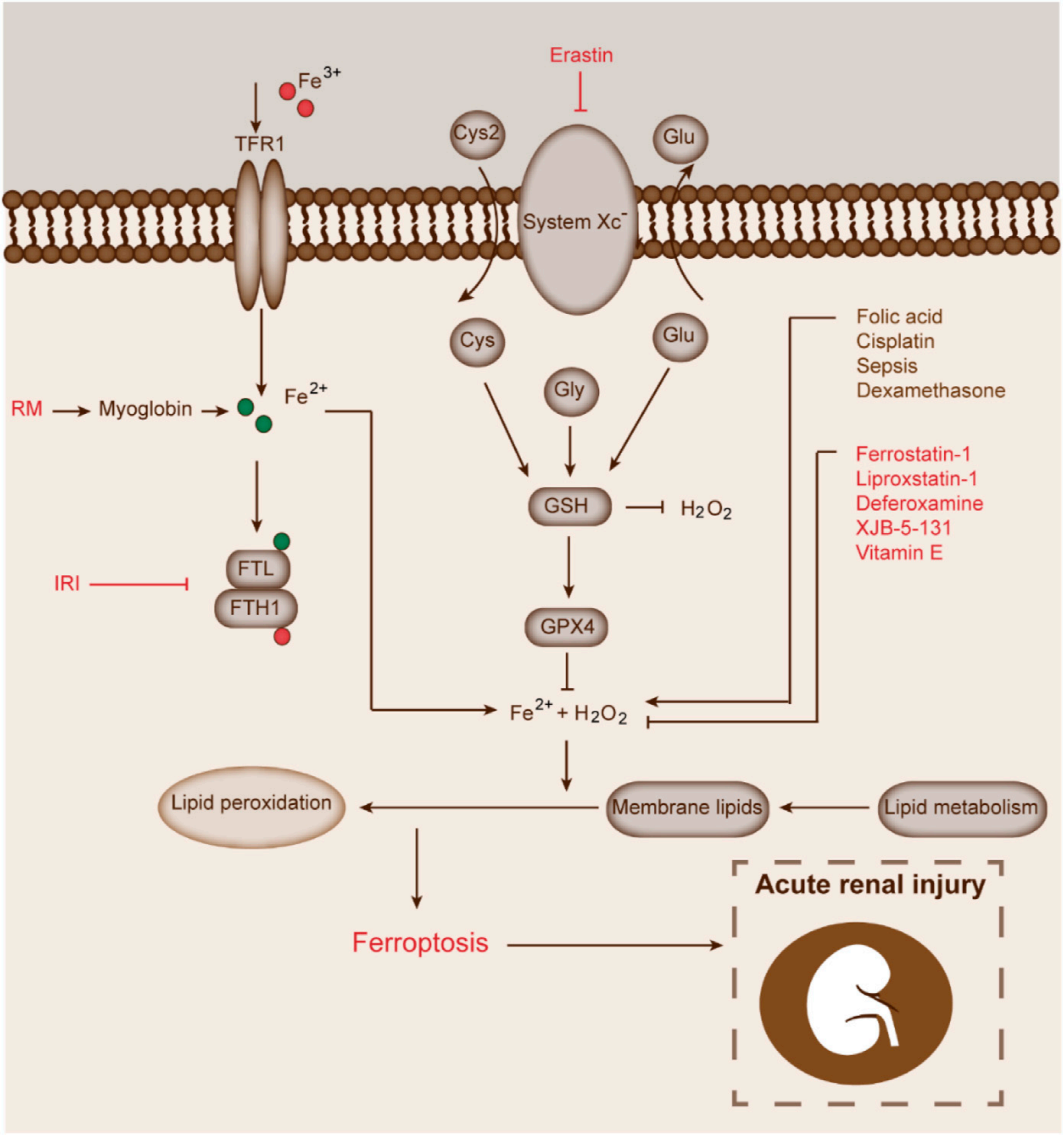
Treatment of AKI by targeting ferroptosis of renal tubular epithelial cells. This figure shows that abnormal increase of Fe^2+^ and H_2_O_2_ promotes the Fenton chemical reaction and lipid peroxidation, and mediates ferroptosis, thus leading to AKI. RM initiates ferroptosis by increasing the levels of Fe^2+^; IRI induces ferroptosis by suppressing the transformation of Fe^2+^ to Fe^3+^. Other pathogenic factors such as FA, cisplatin, sepsis, and dexamethasone can also induce ferroptosis and lead to AKI. Ferrostatin-1, liproxstatin-1, deferoxamine, XJB-5-131, and vitamin E can alleviate or delay the development of AKI by inhibiting ferroptosis.

Inhibiting ferroptosis of renal tubular epithelial cells could effectively alleviate the progression of renal injury in AKI. With the gradual recognition of the role of ferroptosis in AKI, the treatment of AKI by inhibiting ferroptosis has become a hot topic in the field of nephrology (Figure 4).

At present, the main therapeutic advances include ferroptosis inhibitors, lipid peroxidation pathway inhibitors, iron homeostasis regulators, and ROS generation inhibitors. Skouta et al. demonstrated that Fer-1 decreased ROS levels, reduced lipid peroxides, scavenged oxygen free radicals, and inhibited cell death in diverse disease models including AKI (Skouta et al., 2014). Meng et al. reported that similar to Fer-1, ADAMTS13 could also relieve cisplatin-induced inflammatory response and oxidative stress in AKI mice by regulating the Nrf2-mediated ferroptosis (Meng et al., 2021). Liproxstatin-1 has been considered as another ferroptosis inhibitor via delaying lipid hydroperoxide accumulation (Zilka et al., 2017). Friedmann Angeli et al. found liproxstatin-1 could suppress ferroptosis of human renal tubule epithelial cells in IRI-AKI mouse model (Friedmann Angeli et al., 2014). Ma et al. observed that ferroptosis inhibition by liproxstatin-1 significantly reduced kidney lipid peroxidation and the degree of renal histopathological injury in AKI rats (Ma et al., 2021). Yang et al. found that lysyl oxidase inhibitors inhibited ferroptosis in AKI by blocking lipid peroxidation (Yang et al., 2016). Zhang et al. displayed that liproxstatin-1 alleviated the unilateral ureteral obstruction-induced renal injury in AKI mice by inhibiting the ferroptosis of renal tubular epithelial cells (Zhang et al., 2021). In addition to common ferroptosis inhibitors, several other antioxidants and iron chelators (e.g., vitamin E and deferoxamine) were also observed to inhibit ferroptosis by regulating ROS, iron metabolism, and lipid peroxidation. Xiao et al. indicated that miR-212-5p attenuated neuronal ferroptosis during traumatic brain injury by targeting PTGS2 (Xiao et al., 2019b).

Previously, dexamethasone was used as immunosuppressant for the treatment of inflammatory diseases. A latest in ferroptosis research revealed that dexamethasone significantly promoted the ferroptosis in AKI by increasing GR-mediated dipeptidase-1 expression and decreasing the GSH level, which indicated dexamethasone might be an potential ferroptosis agonist (von Massenhausen et al., 2022).

In addition, with the development of traditional Chinese medicines, several herb components were also confirmed to exert anti-ferroptosis activity and further alleviated renal injury in AKI. Wang et al. demonstrated that quercetin could alleviate kidney injury in IRI- or FA-induced AKI mice by inhibiting ferroptosis alleviating (Wang et al., 2021a). . Cheng et al. showed that vitamin D inhibited ferroptosis by activating the Nrf2/GPX4 pathway in zebrafish liver cells (Cheng et al., 2021). Hu et al. also demonstrated that vitamin D receptor activation attenuated cisplatin-induced AKI by inhibiting renal tubular epithelial cells ferroptosis via targeting GPX4 (Hu et al., 2020).

Currently, several studies on the development of ferroptosis inhibitors has been performed in various AKI animal models, but they still lack clinical application potential. Therefore, clinical research on the prevention and targeted treatment of ferroptosis in AKI should be conducted in the future.

## Conclusion

AKI is a type of kidney disease caused by various pathogenic conditions, such as acute tissue ischemia, toxic substance injury, and immune system disorders (Kellum et al., 2021). In view of the complex pathogenesis of AKI, effective strategies for the prevention and treatment of AKI are still lacking (Mercado et al., 2019). Therefore, in-depth analysis of the pathogenesis of AKI is of great significance to alleviate the renal injury and improve the survival of patients with AKI.

Ferroptosis affects the development of various cancers and nervous system diseases (Zheng and Conrad, 2020). With the deepening of research, ferroptosis has been reported to play an important role in the progression of kidney injury, including various AKI models and CKDs. The pathogenesis of AKI is complicated by apoptosis, necrosis, and other forms of cell death in the pathophysiological process, however, the degree of ferroptosis in AKI is different (Han et al., 2020). In addition, the crosstalk between ferroptosis and autophagy, inflammation, oxidative stress, and other regulatory mechanisms in the progression of kidney injury should be further explored (Hu et al., 2019).

Through summarizing the relationship between AKI and ferroptosis, researchers found that ferroptosis inhibition has renal protective effect in different AKI models, but some questions remain to be answered. For example, the mechanism of ferroptosis triggered by Fe^2+^ and lipid peroxide accumulation remains unknown, and the regulatory mechanism of AKI leading to ferroptosis of renal tubular epithelial cells has not been fully clarified. Moreover, further studies are needed to elucidate the specific involvement of ferroptosis especially when multiple cell death modes coexist in AKI and whether different factors affect it. At present, there are no specific biomarkers of ferroptosis compared with apoptosis (caspase activation) and autophagy (autophagy lysosome formation). Therefore, exploring specific biomarkers of ferroptosis is an urgent direction for the study of ferroptosis (Li et al., 2020b). Although the clinical treatment of AKI by targeting ferroptosis has not been reported, there are several reports on the progress of ferroptosis in other diseases, which supports the potential of ferroptosis as a therapeutic target. For example, Kim et al. found cell ferroptosis occurred in diabetic patient kidney tubules with significant decrease of SLC7A11 and GPX4, and ferroptosis inhibition effectively reduced the renal tubules injury (Kim et al., 2021). Clinically, IRI is the main cause of AKI after cardiac surgery. Choi et al. found the level of iron binding protein was decreased during operation, which indicated the Fe^2+^ released by the body was not treated in time, resulting in the increase of unstable iron pool and induction of kidney damage (Choi et al., 2019). Aside from the classical and reported signaling pathways, are there any other ferroptosis regulatory pathways? How should we apply the research findings of ferroptosis to the clinical treatment of AKI? The above problems need to be solved urgently. Therefore, we need to consider how to carry out research to regulate the progress of ferroptosis to prevent the progress of kidney injury. More comprehensive and in-depth research on ferroptosis in AKI and other renal diseases is needed to expand our knowledge and treatment of renal damage, so as to benefit from future clinical results (Wang et al., 2021b).

Overall, ferroptosis plays a key role in the progression of AKI-induced renal injury. Given that there is no effective treatment for AKI, ferroptosis is expected to become a new target for the treatment of this disease.
